# The status and future of essential geodiversity variables

**DOI:** 10.1098/rsta.2023.0052

**Published:** 2024-04-01

**Authors:** Franziska Schrodt, Grant Vernham, Joseph Bailey, Richard Field, John E. Gordon, Murray Gray, Jan Hjort, Carina Hoorn, Malcom L. Hunter Jr., Jonathan Larwood, Angela Lausch, Manu Monge-Ganuzas, Stephanie Miller, Derk van Ree, Arie Christoffel Seijmonsbergen, Phoebe L. Zarnetske, W. Daniel Kissling

**Affiliations:** ^1^ School of Geography, University of Nottingham, Nottingham NG7 2RD, UK; ^2^ Department of Biology, Anglia Ruskin University - Cambridge Campus, Cambridge, Cambridgeshire CB1 1PT, UK; ^3^ School of Geography and Sustainable Development, University of St Andrews, St Andrews KY169AL, UK; ^4^ Queen Mary University of London, London E1 4NS, UK; ^5^ Geography Research Unit, University of Oulu, Oulu 90570, Finland; ^6^ Institute for Biodiversity and Ecosystem Dynamics, University of Amsterdam, Amsterdam 1000 GG, The Netherlands; ^7^ Department of Wildlife, Fisheries, and Conservation Biology, University of Maine, Maine, USA; ^8^ Strategy and Governance, Natural England, Peterborough, Cambridgeshire PE2 8YY, UK; ^9^ Computational Landscape Ecology, Helmholtz-Centre for Environmental Research – UFZ, Leipzig, Saxony 04318, Germany; ^10^ Geoheritage Commission, Spanish Geological Society, Busturia, Biscay 48350, Spain; ^11^ School of Biology and Ecology; Mitchell Center for Sustainability Solutions, The University of Maine, Orono, ME 04469-5751, USA; ^12^ Geo-engineering, Deltares, Delft 2600 MH, The Netherlands; ^13^ Environmental Economics, Vrije Universiteit Amsterdam Faculteit der Betawetenschappen, Amsterdam, The Netherlands; ^14^ Institute for Biodiversity and Ecosystem Dynamics (IBED), University of Amsterdam, Amsterdam, Noord-Holland 1090 GE, The Netherlands; ^15^ Department of Integrative Biology, Michigan State University, East Lansing, MI 48824-1312, USA

**Keywords:** geodiversity, essential variables, natural resource management, environmental policy

## Abstract

Rapid environmental change, natural resource overconsumption and increasing concerns about ecological sustainability have led to the development of ‘Essential Variables' (EVs). EVs are harmonized data products to inform policy and to enable effective management of natural resources by monitoring global changes. Recent years have seen the instigation of new EVs beyond those established for climate, oceans and biodiversity (ECVs, EOVs and EBVs), including Essential Geodiversity Variables (EGVs). EGVs aim to consistently quantify and monitor heterogeneity of Earth-surface and subsurface abiotic features, including geology, geomorphology, hydrology and pedology. Here we assess the status and future development of EGVs to better incorporate geodiversity into policy and sustainable management of natural resources. Getting EGVs operational requires better consensus on defining geodiversity, investments into a governance structure and open platform for curating the development of EGVs, advances in harmonizing *in situ* measurements and linking heterogeneous databases, and development of open and accessible computational workflows for global digital mapping using machine-learning techniques. Cross-disciplinary collaboration and partnerships with governmental and private organizations are needed to ensure the successful development and uptake of EGVs across science and policy.

This article is part of the Theo Murphy meeting issue ‘Geodiversity for science and society’.

## 1. Introduction and background

**Figure 1.**
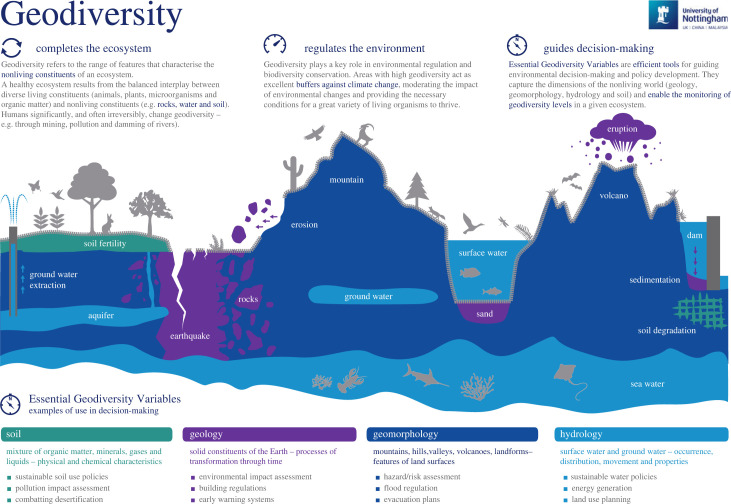
Geodiversity and some of the EGVs with some examples of uses and arguments for importance. Note: this is indicative rather than comprehensive. (Online version in colour.)

Geodiversity, the heterogeneity of the Earth's abiotic surface and subsurface, including geological, geomorphological, hydrological and pedological components [1], is changing rapidly due to processes (e.g. floods, soil erosion, loss of permafrost, terracing, landslides) that are natural but increasingly amplified by climate change, and due to direct anthropogenic activities (e.g. mining, groundwater-extraction, over-exploitation of sand, seafloor trawling, destruction of geoheritage). The need for unified frameworks for efficiently quantifying changes, of both trends and processes, in key aspects of the Earth system, including biodiversity, oceans and climate, led to the development of ‘essential variable' (EV) frameworks (EBVs, EOVs and ECVs, respectively). An equivalent framework for essential geodiversity variables (EGVs) has been established through interdisciplinary research efforts across the fields of hydrology, ecology, geotechnics, conservation biology, climate and environmental science, economics and remote sensing [2]. Understanding of geodiversity is important for (i) geosystem services [3], (ii) conservation of terrestrial, aquatic and marine biodiversity [4], (iii) conservation of geoheritage including its scientific, cultural and other values [5,6], (iv) ecosystem resilience [7], (v) sustainable natural resource development [8], (vi) public health [9], (vii) natural hazard risk management (Geological Society of America) [10], (viii) tourism [11] and (ix) global change [12]. It is highly relevant to policy targets (SDGs, circular economy, energy transition) [13] and the Rights of Nature [14,15]. Furthermore, geodiversity is fundamental to understanding of biotic and abiotic processes and changes throughout the Earth's history [16,17], and thus underlies our ability to understand present-day change and make future projections (figure 1).

Governments, civil society organizations and some commercial companies (e.g. ICT company Nokia; [18]) have also recognized the key role geodiversity plays across society and nature. For example, IUCN Resolution WCC 2020 Res 074 recognizes that ‘selected geodiversity elements and processes, designated as geoheritage, play a crucial role in underpinning the conservation of biodiversity and protected areas, as well as providing other scientific and ecosystem-service benefits' [19]. The UNESCO International Geoscience and Geoparks Programme includes promoting sustainable natural resource use, advancing new initiatives related to geohazards risk mitigation and promoting sustainable development through geotourism and geoparks (https://www.unesco.org/en/iggp). The UN, as well as individual nations and other governing entities, have recognized through policies and law important aspects of geodiversity for the advancement of Earth Jurisprudence (the inherent right of Nature to exist) [20]. For example, two major rivers, the Whanganui River in New Zealand and the Atrato River in Colombia, were granted legal personhood, and the Mar Menor lagoon and its watershed in Spain were granted rights to protection, conservation, maintenance and restoration, plus the right to exist as an ecosystem and evolve naturally [14,15]. Importantly, a combination of indigenous knowledge and scientific understanding of the essential functions of these systems were instrumental in granting protections. In the Te Awa Tupua (Whanganui River Claims Settlement) Act 2017, ‘Te Awa Tupua is an indivisible and living whole, comprising the Whanganui River from the mountains to the sea, incorporating its tributaries and all its physical and metaphysical elements'. Although geodiversity as a term itself has not been directly incorporated into Rights of Nature law or policy, Earth's features and processes have; there is now opportunity to enhance law and policy by incorporating EGVs (see https://ejatlas.org/ for many examples of EGV-related examples).

Schrodt *et al.* [2] first proposed EGVs as a means to unify measurement of, and reporting on, geodiversity features, to highlight the fundamental importance of geodiversity, and to optimize the process from raw data to policy decision relevant metrics. EGVs currently comprise a set of eight measures, namely surface water and groundwater (hydrology), soil chemistry and soil physical state (pedology), landform distribution (geomorphology) and unconsolidated deposits, variability in the intensity of geophysical processes (e.g. volcanic eruptions) and hardrock/mineral/fossil distribution (geology) [2]. Like other EVs, they should be relevant (e.g. for policy), capture states rather than processes, and be feasible and cost-effective to measure repeatedly across space and time. EGVs have been incorporated into the suite of EVs within the Group on Earth Observation Biodiversity Observation Network (GEO BON) (GEO EV report 2023) and their value is recognized as part of the UNESCO International Geodiversity Day.

However, formalizing and operationalizing the EGV framework still requires substantial effort, including both better access to appropriate data and cross-disciplinary work at the science-policy interface—getting the concept into policy documents with legal implications, building capacity and resources for implementation as well as better engaging practitioners. Below, we discuss key barriers and offer potential solutions towards the realization of the EGV framework across disciplines.

## 2. Key questions and challenges

A key challenge for EGVs is that key components of geodiversity have not been integrated into policy and scientific research. One reason for this is that current approaches for identifying, measuring and monitoring geodiversity are inconsistent both between and within disciplines. Monitoring of geodiversity is not explicitly addressed in many spatial-environmental policies, thereby omitting important geosystem services and functions which stem from Earth's geodiversity. Lack of specific analysis of the availability, scarcity and renewability of natural resources when many spatial-environmental policies were developed results in significant threats to geodiversity. Collecting data on EGVs should thus not be seen as mere mapping exercises, which would risk fine-scale but important geodiversity getting lost in classification, averaging and statistical treatment of data—placing important aspects of sustainable management at risk of being missed. Thus, two major challenges for developing policy, and for decision-making based on future scenarios, are (i) scaling up local studies in both space and time and (ii) recognizing the importance of, and accessing data on, fine-scale geodiversity.

In using EGVs, it is important to better quantify environmental and societal impacts of change in the quantity or quality of geodiversity variables and geo-processes and identify possible pathways for resource management and their consequences through integrated Earth System Models such as Digital Twins. This also requires understanding of the link between EGVs and society's socio-economic dimensions (using environmental economic approaches) and capturing potential consequences for both human wellbeing and Earth's Jurisprudence.

## 3. Overlap with other essential variables

While it is encouraging to see how many EVs are currently being developed and operationalized, overlap between them is both a challenge and an opportunity. For example, there is considerable overlap between ECVs and EOVs (28%) and EBVs have a strong interconnection with EOVs (85%) [21]. EGVs such as soil moisture and glaciers are currently listed as ECVs and the EGV structural and benthic complexity are also EOVs. Sometimes, this overlap is superficial, with variables being defined with different resolution, accuracy, latency, length of record and geographical scale needed to fulfil the requirements of different EVs. However, actively examining and potentially coordinating these overlapping EVs can reduce costs and logistical requirements associated with data collection across EV types.

## 4. Semantics and typology of essential geodiversity variables

One of the most fundamental aspects in addressing the challenge of establishing EGVs and incorporating geodiversity into policy is the prevailing lack of consensus on defining geodiversity. This issue has recently been raised [22,23]. Although the prevailing definitions used in geodiversity research follow the one proposed by Gray [24] (see also above), there is also substantial variation. Often, only some of the aspects from the Gray [24] definition are considered (e.g. topography) or non-geodiversity aspects are included within the geodiversity framework (e.g. biotic, climate). This lack of consensus not only makes it more difficult to synthesize findings across studies, it also risks hindering progress and acceptance of EGVs across disciplines. Consistent terminology should be ensured when an EGV is accepted by the scientific community (i.e. any mismatches in a proposed variable are discussed and harmonized where possible). Although this issue is by no means unique to geodiversity, given the breadth of EGVs, covering several scientific disciplines and different pre-existing international definitions (e.g. soil classification systems differing considerably between countries), it is a particularly challenging one.

Developing an openly accessible system for proposing and selecting EGVs—including agreement on semantics—is a critical initial step toward global collaboration and collection of recurring data. For essential climate variables (ECVs), the climate community established a comprehensive list of 47 ECVs through the Global Climate Observing System [25]. Each of these variables was selected based upon an assessment of technical and economic feasibility and is now recognized by the United Nations Framework Convention on Climate Change [25]. However, there is currently no equivalent prevailing system for curating the development of EGVs. One solution could be to expand existing EV inventories already established by organizations such as the Group on Earth Observations (GEO). GEO currently hosts the Global Earth Observation System of Systems (GEOSS) as well the GEO Essential Variables (GEO-EV) Pilot Initiative to foster data integration and harmonization across EVs [26,27]. The interdisciplinary nature of GEO provides the added benefit of limiting the overlap between EVs listed in multiple EV types (e.g. surface variables found in both EGVs and ECVs). This could reduce the overall costs for EV development. GEO already maintains multiple variables associated with geodiversity (e.g. mineral resources and soil properties), but the current readiness level of these variables is reported to be low [21].

## 5. Measuring and accessing data

**Figure 2.**
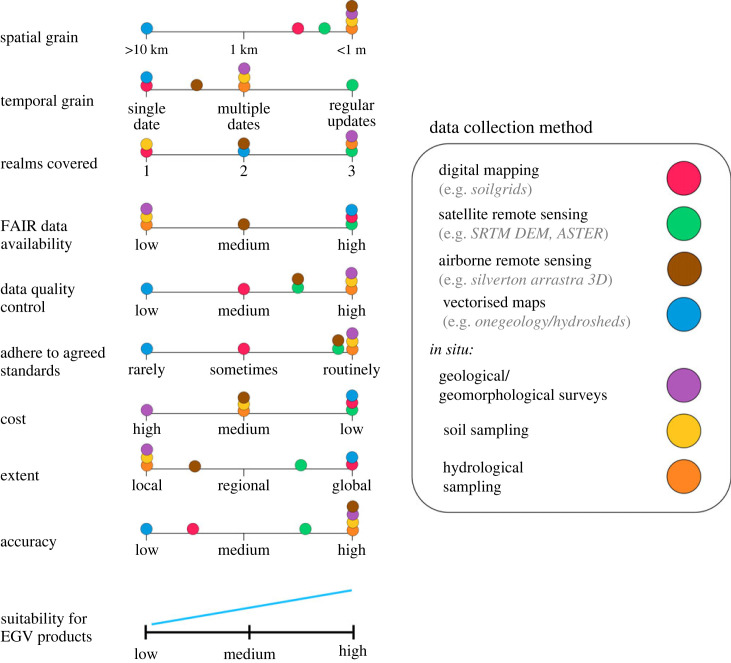
EGV data collection methods mapped against key quality indicators for their use in an essential variables framework. Ideally, criteria should score medium to high in suitability for EGV products (adapted from [35]). SRTM refers to Shuttle Radar Topography Mission. ASTER refers to the Advanced Spaceborne Thermal and Reflection Radiometer. (Online version in colour.)

With the automation of many EGV-relevant *in situ* measurements (e.g. water quality (European Environment [28])) and rapid development of remote sensing-based approaches (e.g. soils [29]), the amount and quality of geodiversity data with higher geospatial and temporal resolution are rapidly increasing. However, some key challenges remain. One is that, although the number of geodiversity components observable directly through remote sensing is increasing (e.g. related to soils [29], landforms [30,31] and hydrology [32]) *in situ* measurements are likely to continue playing a key role in broad-scale geodiversity assessments—perhaps even more than for other EVs. However, we lack harmonization of such measurements, including lack of agreements on standardized protocols for *in situ* measurement of geodiversity (but see [33]). On the other hand, much EGV-related work will by necessity not involve new *in situ* assessments of geodiversity but rather depend on already available data. Many geodiversity databases are now available at local and global scales (e.g. [34]). However, both *in situ* data and established databases do not always follow FAIR (findable, accessible, interoperable and reusable) and CARE (collective benefit, authority to control, responsibility and ethics) data principles (figure 2). Moreover, free and open computational workflows which facilitate accessible means to transform raw geodiversity data into EGVs and higher-level integrators relevant to management and policy are largely lacking. Both have potential for wider adaptation and promotion within EGV relevant disciplines. Below we provide an overview of some challenges and potentials with regards to local/national data products, global data, data accessibility and analysis tools.

### (a) Local to national data

While many local, regional and national databases relevant to EGVs exist, access can be difficult. This is due to three key reasons: (i) many of these data are not easily accessible (e.g. through an open online portal); (ii) expert knowledge is often required to access and use these data, with challenges including judging quality, need for transformations or foreign language skills, and lack of prescriptive metadata and (iii) very high resolution local data are often classified and/or behind paywalls. Large databases also tend to consist of ad-hoc post-hoc sampling rather than systematic measurement of geodiversity with the clear aim of informing EGVs, resulting in strong spatial and temporal biases (figure 2).

EGV data must be relevant, feasible and cost-effective to collect, so that sampling and data collection can be replicated consistently elsewhere. For instance, a very expensive national river flow database may not be feasible for some countries and different measurement approaches will be necessary for meandering, seasonal and braided rivers. By definition, variables should be scalable and suitable for global implementation to be considered as an EV. An efficient means of addressing country-specific differences in the availability of EGV data is the provision of better scaling tools and dedicated research into scaling of EGVs. This would both incentivize countries with higher-quality databases to participate in simpler, global definitions and assessments, as well as outlining which aspects of EGVs may be inferred through scaling and which require in-depth *in situ* measurements for global characterization.

While remote sensing certainly provides a vast improvement and opportunity for local assessment of some geodiversity measures at reduced cost and higher geospatial and temporal resolution, it also comes with challenges. One is, similarly to the *in situ* data discussed above, an issue of data access. Many locally measured data (e.g. through proximal or airborne remote sensing) are not publicly available or are limited to raw formats that require extensive expert knowledge. In other words, a FAIR and CARE data sharing system for local remote sensing data is missing.

Ultimately, we lack a searchable catalogue of databases, facilitating finding, accessing and evaluating local geodiversity data globally and across disciplines. Where networks for consistent monitoring exist, there are missed opportunities resulting from too little coordination between geo-databases and databases of relevant biological data. For example, there are often no auxiliary environmental metadata available in biological databases, despite researchers often measuring at least some geodiversity aspects such as soil characteristics together with their biological data (e.g. TRY, the largest global database of plant functional traits [36] has no environmental metadata associated with it). This is also the case vice versa with most environmental databases having no or little biological metadata available (e.g. only 20% of Fluxnet sites have species data and fewer have traits associated with them). This is largely due to most inventories not being designed to monitor geodiversity (or indeed biodiversity) change using the most sophisticated models and analysis tools or being interoperable with other ecological databases; instead, they comprise *ad hoc* collations of data from disparate sampling campaigns. Relatedly, observation networks often have disparate aims because they have been established by different disciplines with different research questions. A key challenge at the heart of these issues is limited long-term financial support to build interoperable data infrastructures and sustain interdisciplinary groups working on setting up such networks and databases.

While the focus of EGV data is on global availability, monitoring small natural features and geosites could be useful and indeed essential for conserving specialist species and other ecosystem properties [37–39]. The feasibility of monitoring such features globally is challenging given current technological constraints. However, it is important to recognize the key function of such small features in global geodiversity assessments and further promote research in improving sensing capabilities, e.g. building on developments in remote sensing combined with AI to automatically classify small natural features over large areas [40].

### (b) Global data

Earth observation techniques are developing rapidly and there is a growing demand for remotely sensed data as improvements in spatial and temporal resolution allow for a wide range of physical properties to be captured (table 1 for a non-comprehensive list of available global-scale EGV-relevant data; see also https://bioxgeo.github.io/bioXgeo_ProductsTable/ from [41]). Such products can be a rich source of recurring geodiversity data from continental to global extents. The widespread availability of digital elevation, surface and terrain models (DEMs, DSMs and DTMs, respectively) illustrates this trend, with high-resolution global models now openly available (e.g. 30 m global DEM from the Shuttle Radar Topography Mission). These data can be used to generate a wide range of terrain attributes (e.g. geomorphological features [42] and wetness indices [43]) which can then be processed to model geodiversity patterns on a continuous surface globally. However, the relevance of certain terrain attributes to Earth's socio-ecological systems (e.g. ecosystem function) is an area in need of further research to assess their essentiality within EGV frameworks. Other remotely sensed geo-physical products have also been developed. For example, the Advanced Spaceborne Thermal Emission and Reflectance Radiometer (ASTER) has produced high-resolution maps (15–90 m) on lithology and superficial deposits, providing a promising source for geological EGVs globally.

**Table 1.** Non-comprehensive list of data sources for each EGV class with key criteria. Note: the focus is on global/continental and open access data.

	data availability			
EGV class	data source	realm covered	spatial resolution	temporal information/resolution
**geology**				
rock types (lithology classes)	https://www.geo.uni-hamburg.de/geologie/forschung/geochemie/glim.html	terrestrial	polygons (average map scale: 1 : 3 750 000)	unclear
rock types (lithology classes combined with ecosystems)	https://zenodo.org/record/1464846#.Xn3P40p7lPY	terrestrial	250 m	2014
terrestrial sediments	https://doi.pangaea.de/10.1594/PANGAEA.884822	terrestrial	polygons (average map scale 1 : 3 000 000)	unclear
OneGeology	https://portal.onegeology.org/OnegeologyGlobal/	terrestrial (global), marine (some Europe and USA only)	1 : 3 M (marine), 1 : 50 M (terrestrial)	unclear
marine geology	https://www.ngdc.noaa.gov/mgg/geology/geology.html	marine	various	unclear
marine geology (continental shelf)	http://maps.continentalshelf.org/	marine (continental shelf)	point data	sampling date
**hydrology**				
surface waters	https://global-surface-water.appspot.com/	freshwater and marine	30 m	different number of observations between March 1984 and October 2015
streamflows	https://www.nature.com/articles/sdata201852	freshwater	∼1 km	1960–2015
rivers and catchments	https://www.hydrosheds.org/pages/gloric	freshwater	∼500 m (no coverage above 60° northern latitude)	unclear
rivers and catchments	https://www.hydrosheds.org/hydrosheds-v2	freshwater	∼12 m	unclear
wetlands	https://doi.pangaea.de/10.1594/PANGAEA.892657	freshwater and coastal	∼500 m	unclear
**soil**				
soil properties and classes	https://soilgrids.org/	terrestrial	250 m	snapshot (September 2019)
USDA soil great groups	https://zenodo.org/record/3528062#.XnNGCUp7lPY	terrestrial	250 m	different sampling times 1950–2017
**geomorphology**				
geomorphometry	https://peerj.com/preprints/27595/	terrestrial	90 m	unclear
landforms (7 classes)	https://zenodo.org/record/1464846#.Xn3P40p7lPY	terrestrial	250 m	2014
landforms (10 classes)	http://www.earthenv.org/topography	terrestrial	from 1, 5, 10, 50 to 100 km spatial grains	combination of 2003 and 2010 data
bathymetry	http://maps.continentalshelf.org/	marine (continental shelf)	point data	sampling date
ocean floor geomorphology	blue habitats—home to the global seafloor geomorphic features map	ocean floor, shelf	vector data	unclear
**geodiversity**				
global geodiversity layers	https://doi.org/10.21942/uva.23496923	global	10 × 10 km	static, various dates

Digital mapping using machine-learning techniques to interpolate geodiversity properties from available environmental data has also been employed, most pervasively in soil mapping. For example, the SoilGrids dataset provides global coverage of multiple soil properties at 250 m resolution [44]. While these maps are trained using *in situ* soil measurements, such *in situ* data tend to be sparse, particularly outside European and Northern American areas. Thus, the interpolation of properties using machine learning introduces sometimes considerable uncertainty, reducing reliability in data-poor areas such as the Arctic, which should be considered prior to inclusion as EGV data sources. In other words, uncertainty needs to be quantified for each EGV data layer to allow data users to make informed decisions (based on their specific application and expertise) on how much uncertainty is acceptable or, even better, enable propagation of uncertainties through analyses (e.g. within a Bayesian framework).

The coarse resolution of global digital soil maps (relative to other geodiversity datasets such as DEMs) may limit their relevance as useful EGVs at global scales. Currently, finer-resolution digital soil maps are limited to national extents (e.g. 25 m digital soil map of Switzerland; [45]), though this is likely to improve in the near future, particularly due to increased availability of remote-sensing techniques to map pedological properties [29].

While digital mapping does provide a cost-effective way of modelling many abiotic properties, it does treat dynamic properties statically and thus requires recurring recalibration as environmental covariates change, combined with recurring *in situ* validation efforts to ensure a suitable level of accuracy across digitally mapped EGVs. This will affect some geodiversity aspects more than others. Static data are appropriate where the location and state of EGVs is changing very slowly (e.g. bedrock type) but other EGVs will require frequent and consistent monitoring through time at yearly (e.g. geomorphological processes such as slope stability and surface materials) or even seasonal or daily time scales (e.g. monsoon-related hydrological networks, resource extraction such as sand mining and soil characteristics). Further, it is important to consider potential circularity due to covariates (e.g. climate, vegetation cover) being used in the modelling/extrapolation to derive global maps.

Vectorized maps of many geological, hydrological and geomorphological properties (e.g. bedrock type) are available at global scales (e.g. OneGeology, Global unconsolidated sediments map and WWF HydroSHEDS). These vectorized maps are generally accessible and harmonized with clear supporting metadata describing aggregation and vectorization methods. However, the usefulness of these datasets as source data for EGVs remains largely unknown. The sometimes limited spatial resolution and static nature of these datasets may render them ineffective for informing policy and monitoring socio-ecological change and practice and shows that their content and usefulness differs depending on data requirements. For example, Fleischer *et al.* [46] analysed several databases (maps) covering European surface and bedrock geology, sediment thickness and structural information. In a first-time practical application, they applied OneGeology and showed clear limitations in terms of providing depth-resolved data. Thus, with respect to geodiversity and provision of geosystem services, there is a significant challenge in both developing and filling a coherent database (such as OneGeology) but also in addressing shortcomings that evolve in applying and evaluating the data. Particularly, the three-dimensional aspect is a requirement that provides challenges going beyond superficial characteristics. However, there is opportunity to integrate satellite remotely sensed data with vectorized data to produce derived data products. For example, the combination of ASTER and Macrostrat, which includes global spatial and temporal data for hard rocks (igneous, metamorphic, sedimentary; [47]), could produce more comprehensive geological EGVs globally. Assessing lag and legacy effects requires long-term temporal data and a high temporal grain of global databases—an aspiration we are inching closer to.

### (c) Data access and analysis tools

While both local and global geodiversity data are challenging but increasingly available, the same applies to tools which facilitate access, evaluation and modelling of EGV-relevant data. For example, the Geodoc Metadata Editor facilitates the creation, validation, editing and export of geospatial metadata and ensures compliance with major data standards. The FAIRMetrics tool facilitates assessment of the FAIRness of a digital resource (table 2).

**Table 2.** Key tools facilitating good practice in EGV data recording, storage and distribution, including adherence to relevant standards and semantics.

tool	purpose	link
ANZ-MEST	create, validate, edit and export geospatial metadata records. Creation of records as XML output files compliant with many relevant standards	https://www.anzlic.gov.au/resources/anzlic-metadata-toolkit
FAIRMetrics tool	assess the FAIRness of a digital resource	https://github.com/FAIRMetrics/Metrics
DDI tools	Data Documentation Initiative website's list of tools to implement the DDI standard	https://ddialliance.org/resources/tools
DDI on Rails	software for building a data portal, with a particular focus on survey datasets	https://www.impactdistillery.com/ddionrails
Geodoc Metadata Editor	facilitates creation, validation, editing and export of geospatial metadata records as XML output files compliant with a number of standards	https://www.geoportal.sk/en/aplikacie/metadata-editor/
ESIS (Ecosystem Integrity - Remote Sensing/Modelling Service)	facilitates derivation of remotely sensed indicators to quantify geodiversity traits (also vegetation and land use intensity)	https://zenodo.org/record/8116370
Dataverse	an open-source web application to share, preserve, cite, explore and analyse research data	https://dataverse.org/

Raster datasets derived from remote sensing (e.g. DEMs) can be analysed using algorithmic functions applied to pixel values or segmented to objects grouped from adjacent properties to create new datasets. This is especially relevant to geodiversity variables that aim to capture the heterogeneity of abiotic components. Information theory techniques have been used to develop geospatial software (e.g. rasterdiv; [48]) that allows raster datasets to be analysed for heterogeneity values across large extents in a reproducible manner. These tools can be applied to any continuous raster map (e.g. terrain attributes or digital soil maps) to compute features of geodiversity. For example, elevation-based heterogeneity rasters (e.g. terrain ruggedness; [49]) are heavily used in geoscientific and environmental research. However, more research is required to assess the value of heterogeneity rasters across geodiversity components (e.g. soil properties and landform types) as EGVs, particularly in the context of supporting biodiversity conservation, geosystem services, natural hazard risk management and ecosystemfunction.

## 6. Ways forward

### (a) Semantics and typology of essential geodiversity variables

One potential area of concern that has emerged in geodiversity research is coming to a consensus on a single definition for geodiversity ([23] in review). While researchers and practitioners have largely accepted the definition used here [1], there is still some disagreement over which abiotic elements and groups should be considered components of geodiversity. This can create confusion over which components should be assessed for feasibility and inclusion as EGVs. We suggest using this established definition of geodiversity to avoid confusion, as EGV frameworks and research agendas are developed. Furthermore, we would incentivize the use of established terminologies when engaging in discussion, collaboration and research on EGVs, and call for the establishment of an international glossary to facilitate this process (e.g. following the example of the WMO international glossary of Hydrology). The fields of geological, geomorphological, hydrological and pedological research have a long history, and using established terms will aid in clear discussion between experts (e.g. [50]).

### (b) Measuring and accessing data

The establishment of international and national platforms to host community discussion, research and data integration to inform selection of feasible EGVs is a logical first step towards reaching a point where EGV data can be collected and monitored on a recurring basis. As acquiring and maintaining long-term funding for a dedicated data platform is challenging, open-access data-deposition platforms already tailored toward hosting data on EVs are an obvious starting point. For example, GEO hosts a large cohort of existing EVs, some of which serve as components of geodiversity (topographic and soil properties). It is already involved in published research linking geodiversity to biodiversity (e.g. [30]). Furthermore, GEO maintains active national partnerships with governmental and private organizations with specialist expertise in geospatial modelling, remote sensing, environmental modelling and high-powered computing. GEO also hosts integration of many regional platforms, most notably from Copernicus (e.g. ArcticGEOSS platform), which provide opportunities for remotely sensed data integration in EGV development. The platform is thus ideal for research sharing, collaboration and forum discussion on EGVs. However, the concept of EGVs currently remains unlisted. Another potential platform is OpenGeoHub, which has been developed to advance intuitive, open-source data focused toward combining remotely sensed data with *in situ* measurements, e.g. for soil, climate and topographic data layers (e.g. MODIS water vapour 1 km). Other potential international data platforms could be the newly established ESIS tool (https://zenodo.org/record/8116370) or the World Environment Situation Room (https://wesr.unep.org/).

Ultimately, as different EVs serve different communities and will be maintained by different organizations and experts, the key is data and technical interoperability across infrastructures and different platforms [51]. Interoperability is defined as ‘the capability to communicate, execute programmes or transfer data among various functional units in a manner that requires minimal knowledge of the unique characteristics of those units' (ISO/IEC 2382:2001 Information Technology Vocabulary – Fundamental Terms) or, more succinctly as ‘the ability of two or more systems or components to exchange information and to use the information that has been exchanged' [52]. Many tools are now available to facilitate this (table 2), and frameworks towards achieving full interoperability are accessible [51].

Following the establishment of EGVs on an international platform, the next challenge is to build a forum that incentivizes discussion and assessment among experts on the feasibility of geodiversity variables for selection of EGVs, and moreover, agreement on best practices to harmonize techniques for recurring data collection of selected EGVs and optimization of sampling methodologies. Such an agreement can be achieved through integrated scientific and technical workshops encouraging open cooperation between those collecting, processing and storing data necessary to characterize selected EGVs. This is comparable to the strategy used for widespread establishment of ECVs through the Global Climate Observing System and should aim to:

1. Identify potential EGVs beyond those identified in Schrodt *et al.* [2] and assess them for essentiality (e.g. effectiveness and complementarity), feasibility (i.e. technology and costs), unambiguity (e.g. accuracy and resolution) and evolvability (e.g. consensus) [53].
2. Establish techniques for data collection, integration and preservation.
3. Ensure data standards to promote compatibility and comparability (table 3).

**Table 3.** Non-comprehensive list of some of the key data standards relevant for the development of EGVs.

standard	details	source	geology	geomorphology	hydrology	soils
OGC API - Environmental Data Retrieval Standard	a family of lightweight query interfaces to access spatio-temporal data resources by requesting data at a position, within an area, along a trajectory or through a corridor	https://docs.ogc.org/is/19-086r6/19-086r6.html	X	X	X	X
OGC Cloud Optimized GeoTIFF Standard	formalizes the requirements for a TIFF file to become a Cloud Optimized GeoTiff (COG) file and for the HTTP server to make COG files available in a fast fashion on the web	https://docs.ogc.org/is/21-026/21-026.html	X	X	X	X
ISO 19115	provides information about the identification, the extent, the quality, the spatial and temporal schema, spatial reference and distribution of digital geographical data	https://www.iso.org/standard/53798.html	X	X	X	X
INSPIRE Metadata Regulation	a profile of ISO 19115:2003, adopted in 2007 as the common metadata standard for the Infrastructure for Spatial Information in the European Community (INSPIRE)	https://inspire.ec.europa.eu/	X	X	X	X
GeoSciML	defines a model and the encoding for geological features commonly described and portrayed in geological maps, cross sections, geological reports and databases	http://geosciml.org/	X			
Dublin Core	a basic, domain-agnostic standard which can be easily understood and implemented, and as such is one of the best known and most widely used metadata standards	https://www.dublincore.org/	X	X	X	X
Data Documentation Initiative (DDI)	a widely used international standard for describing data from the social, behavioural, and economic sciences	https://ddialliance.org/	X	X	X	X
Darwin Core Geospatial Extension	provides a protocol-independent XML schema for a geospatial extension to the Darwin Core	https://github.com/tdwg/wiki-archive/blob/master/twiki/data/DarwinCore/GeospatialExtension.txt	X	X	X	X
European Directory of Marine Environmental Data (EDMED)	European standard for indexing and searching datasets relating to the marine environment.	https://www.bodc.ac.uk/resources/inventories/edmed/	X	X	X	
OGC WaterML 2: Part 3 - Surface Hydrology Features (HY_Features)	Defines a common conceptual information model for identification of specific hydrologic features. Includes relationships such as hierarchies of catchments, segmentation of rivers and lakes, and the hydrologically determined topological connectivity of features such as catchments and waterbodies. Independent of their geometric representation and scale.	https://docs.ogc.org/is/14-111r6/14-111r6.html			X	
Soil data standard complication	outlines commonly used soil data related standards with different levels of recommendation/readiness	Hoffmann *et al.* [54] Data Standards for Soil- and Agricultural Research. BonaRes Series 2019/6. doi:10.20387/BonaRes-ARM4-66M2				X
Open Geospatial Consortium	provides a comprehensive list of major data standards for geospatial data, across EGV classes	https://www.ogc.org/standards/	X	X	X	X
Bath Metastandards Catalog Geomorphology	provides a list of data standards relevant to geomorphology	https://rdamsc.bath.ac.uk/subject/Geomorphology		X		
Bath Metastandards Catalogue Hydrology	provides a list of data standards relevant to hydrology	https://rdamsc.bath.ac.uk/subject/Hydrology			X	

A promising alternative approach the geo-community could adapt has been developed for the biodiversity community with BON in a box. This is a free online system aimed at facilitating networking, calculation of Essential Biodiversity Variables using data linked to BON in a box, optimizing monitoring using that same data and joined reporting of indicators to monitor global progress [55].

### (c) Unrealized (or under-used) sources of data

Finally, we call for further cross-disciplinary collaboration and integration of under-used data sources which could be relevant to EGVs. These include making better use of the large amount and increasing quality of citizen and community science data available (e.g. as implemented for soil data [56]). These data will need to be carefully evaluated. For example, a study of the use of citizen science data for evaluating geodiversity across different national parks in Poland found little overlap between expert and volunteer evaluation in parks with less geomorphological diversity, and relatively high quality of non-expert evaluation in geomorphologically diverse areas [57]. We also suggest facilitating access for scientific purposes to commercial EGV-relevant data. For example, numerous soil testing laboratories carry out regular soil tests using standard methodologies across the world, resulting in vast amounts of geospatial and temporally explicit data which, however, are largely not accessible for research. Tapping into, or establishing, global sensor networks such as the very successful and rapidly growing soil temperature database [58] is another promising avenue. Finally, further automating collection of *in situ* data would allow us to fill some of the many blank areas in the map of EGV data. Particularly useful for data collection in remote locations are new proximal remote sensing technology and biodegradable sensors [59].

## 7. Conclusion

In order to effectively assess the likely consequences on ecosystem and geosystem services, biodiversity and human wellbeing resulting from changes in geodiversity globally, the transdisciplinary approach we propose is urgently needed. Ultimately, we as a community of data producers, modellers and data/information users need to agree on which geodiversity measures are essential (relevant, feasible, cost-effective), what we can usefully measure (with usefulness spanning many aspects, including policy, society, biodiversity, geoheritage and Earth jurisprudence) at the global scale in a cost-effective way, and how best to facilitate access and reuse. This will help establish an active, long-term platform ensuring maximum transdisciplinary exchange and effective development of a consistent, well-defined set of EGVs that will be useful in science, policy and society.

## Data Availability

This article has no additional data.
